# Correction: Paclitaxel prodrug based mixed micelles for tumor-targeted chemotherapy

**DOI:** 10.1039/d0ra90029j

**Published:** 2020-04-02

**Authors:** Dongyang Tang, Xin Zhao, Tie Yang, Cheng Wang

**Affiliations:** Department of Experimental Center, Henan Institute of Science and Technology Xinxiang Henan 453003 P. R. China; Department of Pharmacy, Xinxiang Central Hospital Xinxiang Henan 453000 P. R. China; Nanjing Research Center, Jiangsu Chiatai Tianqing Pharmaceutical Co. Ltd Nanjing Jiangsu 210042 P. R. China; College of Pharmaceutical Sciences, Zhejiang University 866 Yuhangtang Road Hangzhou Zhejiang 310058 P. R. China 11519016@zju.edu.cn

## Abstract

Correction for ‘Paclitaxel prodrug based mixed micelles for tumor-targeted chemotherapy’ by Dongyang Tang *et al.*, *RSC Adv.*, 2018, **8**, 380–389.

The authors regret that in Fig. 4C, the image of the SM group with FA pretreatment was mistakenly a repeated version of the image of the SM group without FA pretreatment. The correct figure is included in this correction. This correction does not affect the scientific validity of the article.

**Fig. 4:**
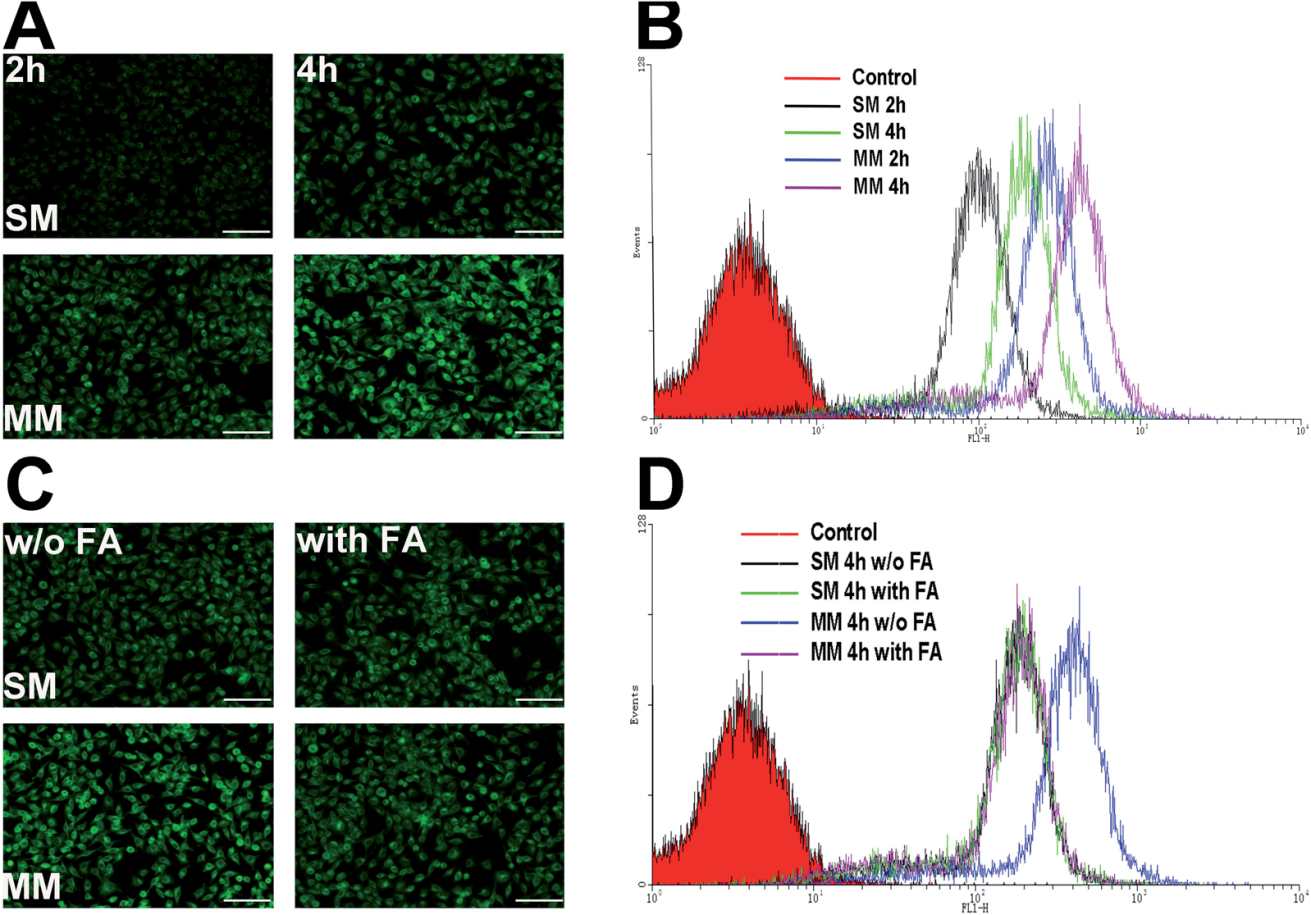
The *in vitro* cellular uptake analysis of SMs and MMs in the Hela cells. (A) The inverted fluorescence microscope images of the cells incubated with the C6-loaded SMs and MMs for 2 and 4 h. Scale bar: 200 μm. (B) Flow cytometric analysis of mean fluorescence intensity in the cells treated with the C6-loaded SMs and MMs for 2 and 4 h. (C) Inverted fluorescence microscope images of the cells incubated with the C6-loaded SMs and MMs with or without the FA pretreatment 4 h. Scale bar: 200 μm. The data are shown as mean ± S.D. (*n* = 3). (D) Flow cytometric analysis of mean fluorescence intensity in the cells treated with the C6-loaded SMs and MMs with or without the FA pretreatment 4 h.

The Royal Society of Chemistry apologises for these errors and any consequent inconvenience to authors and readers.

## Supplementary Material

